# Widening mismatch between UK seafood production and consumer demand: a 120-year perspective

**DOI:** 10.1007/s11160-023-09776-5

**Published:** 2023-06-02

**Authors:** Luke O. J. Harrison, Georg H. Engelhard, Ruth H. Thurstan, Anna M. Sturrock

**Affiliations:** 1 School of Life Sciences, University of Essex, Wivenhoe Park, Colchester, CO4 3SQ UK; 2 Centre for Environment, Fisheries and Aquaculture Science (Cefas), Pakefield Road, Lowestoft, NR33 0HT UK; 3 School of Environmental Sciences, University of East Anglia, Norwich, NR4 7TJ UK; 4 Centre for Ecology and Conservation, University of Exeter, Penryn Campus, Cornwall, TR10 9FE UK

**Keywords:** Aquaculture, Food security, Historical ecology, International trade, Policy change, UK fisheries

## Abstract

Developed countries are increasingly dependent on international trade to meet seafood requirements, which has important social, environmental, and economic implications. After becoming an independent coastal state following Brexit, the UK faces increased trade barriers and changes in seafood availability and cost. We compiled a long-term (120-year) dataset of UK seafood production (landings and aquaculture), imports, and exports, and assessed the influence of policy change and consumer preference on domestic production and consumption. In the early twentieth century, distant-water fisheries met an increasing demand for large, flaky fish such as cod and haddock that are more abundant in northerly waters. Accordingly, from 1900 to 1975, the UK fleet supplied almost 90% of these fish. However, policy changes in the mid-1970s such as the widespread establishment of Exclusive Economic Zones and the UK joining the European Union resulted in large declines in distant-water fisheries and a growing mismatch between seafood production versus consumption in the UK. While in 1975, UK landings and aquaculture accounted for 89% of seafood consumed by the British public, by 2019 this was only 40%. The combination of policy changes and staunch consumer preferences for non-local species has resulted in today’s situation, where the vast majority of seafood consumed in the UK is imported, and most seafood produced domestically is exported. There are also health considerations. The UK public currently consumes 31% less seafood than is recommended by government guidelines, and even if local species were more popular, total domestic production would still be 73% below recommended levels. In the face of climate change, global overfishing and potentially restrictive trade barriers, promoting locally sourced seafood and non-seafood alternatives would be prudent to help meet national food security demands, and health and environmental targets﻿﻿.﻿

**Supplementary Information:**

The online version contains supplementary material available at 10.1007/s11160-023-09776-5.

## Introduction

Seafood represents a critical source of protein and micronutrients to billions of people globally (FAO 2022). International trade of fish and fish products is increasing rapidly, and seafood is now one of the most traded food products in the world (Asche et al. 2015). While trade occurs in all directions, most imports of high value seafood are from developing countries to developed countries, the costs and benefits of which are hotly debated (Asche et al. 2015). While a thriving export market can provide economic growth and alleviate poverty for example by developing countries using their resources such as labour and natural capital more efficiently based on their comparative advantage (Thorpe et al. 2005; Valdimarsson and James 2001), it can also negatively impact local livelihoods and food security (Abgrall 2003; Alder and Sumaila 2004). Seafood can also be imported and exported for processing several times prior to being consumed (Bellmann et al. 2016; Watson et al. 2016), increasing carbon emissions, and reducing biodiversity via habitat degradation, pollution and spreading of non-indigenous species (Lenzen et al. 2012; Parker et al. 2018; Whittington et al. 2008). The factors driving global seafood trade are still open to debate, however changes in technology, policy and trade regulations have all had major influences. Following World War II, improvements in vessel and fishing gear technology resulted in an expansion of distant-water fisheries, causing global fish landings to rise (Alder and Watson 2007; Kerby et al. 2012; Thorpe and Bennett 2001). From the mid-1970s, countries started to adopt 200 nautical mile Exclusive Economic Zones (EEZ), leading to a decline in many large distant-water fleets, and increased investment in domestic fisheries (Asche et al. 2015; Kerby et al. 2012). The adoption of EEZs indirectly resulted in increased global seafood trade, as countries with excluded distant water fleets compensated their fish requirements with increased imports (Asche et al. 2015; Bellman et al. 2016). Since the 1980s, globalisation and trade liberalisation policies have accelerated growth in global seafood trade (Alder and Watson 2007; Thorpe and Bennett 2001). It is less costly to trade seafood than other food products as the World Trade Organisation classify fish as an industrial product rather than an agricultural product, resulting in lower tariff protection (Bellmann et al. 2016; Pene and Zhu 2021). Stricter fisheries management by developed countries following historic overexploitation of their fish stocks has also created increased local demand and contributed to the rise in trade (Helvey et al. 2017; Klein et al. 2022; Worm et al. 2009). Although these measures have recovered many stocks (Hilborn et al. 2020), the total proportion of overfished stocks continues to rise from 10% in the mid-1970s to 34.2% and 35.4% in 2017 and 2019 respectively, and global landings have plateaued since the 1980s despite increased fishing effort (Bell et al. 2017; FAO 2020, 2022; Watson et al. 2013). Adding complexity to spatial management efforts, fish distributions and abundances are changing rapidly due to warming and habitat change, with many species shifting into higher latitudes and greater depths to meet temperature requirements (Brander 2010; Cheung et al. 2013).

For the UK, seafood trade has always been important, but it has become increasingly debated in recent times after the UK withdrew from the European Union (EU) and Common Fisheries Policy (CFP) in December 2020 (Stewart et al. 2022). Historically, the UK was a major seafood exporter, with most UK landings in the early to mid-twentieth century being from distant-water fisheries, which were largely discontinued following widespread adoption of EEZs (Kerby et al. 2012). In 1973, the UK joined the European Economic Community (EEC) with certain fisheries resources subsequently managed under the CFP (Kerby et al. 2012). The EEC was renamed the European Community (EC) in 1993, which became part of the EU (Treaty on European Union 1992). The CFP introduced regulations such as Total Allowable Catches and national quotas for specific fish species (Kerby et al. 2012). Allocated quotas were partly based on historical catches from areas such as the North Sea, as well as the needs of areas dependent on fishing, and the loss of landings in other countries’ waters following the introduction of EEZs (Kerby et al. 2012). As landings in the North Sea had already declined for the UK due to England’s increased focus on distant-water fishing in previous decades, the UK fleet were allocated low quotas of some fish species (Kerby et al. 2012), although these were partially compensated by higher quota shares of other species, such as mackerel (Hoefnagel et al. 2015; Whitmarsh and Young 1985). The introduction of EEZs and CFP policy have been linked to a decline in UK landings (Kerby et al. 2012), but it is unclear to what extent these policy changes influenced domestic fish consumption and trade.

More recently when the UK left the EU and the CFP, it also left the European Single Market. The European Single Market had previously removed many of the trade barriers between the UK, European states, and countries such as Norway and Iceland (Cabinet Office 2021; Phillipson and Symes 2018). The EU-UK Trade and Cooperation Agreement (2020) outlines the new trade agreement between the UK and EU member states, including no tariffs on traded goods providing they originate in the UK. For seafood, this would include UK aquaculture production and fish caught in the UK’s territorial waters of 12 nautical miles (Seafish 2021). Additionally, the UK signed a Free Trade Agreement with Norway, Iceland, and Liechtenstein (2021), which included tariff reductions for some seafood types. Fish landed by UK and EU vessels outside of their EEZs, however, would not be tariff free as they were under the European Single Market, and stricter custom controls at EU and UK borders have been created, which may affect the trade of perishable seafood (Seafish 2021). Brexit therefore has important implications for the UK seafood industry, as it may affect exports of seafood caught outside of the UK EEZ and the cost and types of fish available to its consumers as the new controls may reduce seafood imports from the EU.

The UK consumes a huge quantity of imported fish and shellfish products (Thurstan and Roberts 2014). Jennings et al. (2016) suggested that such a high reliance on imports leaves the UK open to risks such as fish availability being affected by changes in trade relationships. Indeed, the Brexit vote was an unforeseen risk that may affect UK trade, due to increased tariffs on seafood not caught within the UK EEZ and the introduction of custom controls ﻿(Seafish 2021; Stewart 2022). Heavy reliance on imports also challenges the UK’s commitment to achieving net-zero carbon emissions by 2050, including those from international aviation (UK Government 2021).

Understanding long-term patterns in UK fish trade and availability, and to what extent these patterns were influenced by policy changes, could provide insights into the potential of current policy developments to impact future UK fish availability. Here, we explore the influence of policy change and consumer preferences on the international trade of seafood by the UK over the last 120 years, and how changes in these factors have created a disconnect between the seafood produced and consumed by the UK. Specifically, we examine (1) historical changes in UK seafood production and trade relative to recommended per capita intake levels of seafood, (2) if the money generated by production and exports exceeded the amount spent on imports, (3) changes in the composition of species produced and traded through time, and (4) mismatches between production and consumption through time, and the relative influence of past policy changes and political events.

## Methods

### Seafood production and trade over time

To estimate UK seafood production between 1900 and 2020, the total weight of marine finfish and shellfish landed by UK vessels in domestic ports (domestic landings) was digitised from annual UK Sea Fisheries Statistics tables (MMO 2014, 2021a). UK inland landings were not included, as at their peak in 1999, they only accounted for 0.8% of local UK fish production at 4835 tonnes (FAO 2021a). UK-based aquaculture production estimates were obtained from the Food and Agriculture Organization (FAO) for 1950 to 2019 (FAO 2021b). UK aquaculture production data was not available before 1950 but it was likely negligible based on the low aquaculture production quantity early in the dataset. As the Republic of Ireland left the UK and partitioned from Northern Ireland in 1921 (Castan Pinos and McCall 2021), Ireland was included in all datasets between 1900 and 1921, and only Northern Ireland thereafter. Unless otherwise stated as having been converted into ‘processed weights’ (see below), production was estimated using the weights provided in the original data source, representing total weight for shellfish (which includes crustaceans and molluscs, and molluscs include cephalopods and bivalves) and herring (which are landed whole) and gutted weight for other finfishes. Data sources and handling are fully detailed in Tables S1 and S2, respectively.

Total UK seafood import and export weights were digitised from the same UK Sea Fisheries Statistics (Table S1). Fish meals and oils were excluded, as the majority are used in agriculture and aquaculture rather than directly consumed (Thurstan and Roberts 2014). It is worth noting that imports and exports cannot be treated entirely independently, as some fish are exported for processing then reimported, and vice versa (Jennings et al. 2016).

### Seafood consumption

To estimate the fraction of UK seafood production that is consumed by the UK public, finfish and shellfish weights were adjusted to processed weights using the mean conversion factors described in Thurstan and Roberts (2014) (Finfish; 0.49 ± SE 0.02; Shellfish; 0.28 ± SE 0.05). The conversion factors represent gutted weight (or whole weight for herring) to fillet weight for finfish species, and the edible portion of shellfish. The total weight of seafood consumed each year (‘consumption’) was calculated as total processed landings by the UK fishing fleet (in the UK and abroad) plus processed UK-based aquaculture plus total seafood imports minus all seafood exports out of the UK. Although some fish are traded whole, most are processed prior to trading, e.g., canned, frozen or salted. As such, import and export weights were not corrected for processing. It is also worth noting that some domestic landings are used in agriculture and aquaculture rather than directly consumed, but the fraction is generally low (Green 2012; Tacon and Metian 2008).

To assess whether nutrition requirements could have been met through domestic seafood production, we used the Food Standards Agency recommendation that adults should consume at least two 140 g fish portions per week, including one portion of oily fish (Clonan et al. 2012). The recommended fish intake weight was adjusted to the UK population per year, sourced from the Office for National Statistics (ONS 2021) and the Central Statistics Office of Ireland (CEIC 2020). It was assumed that children under 15 years old on average only need half of the fish intake recommended for adults (Thurstan and Roberts 2014).

### Economics

To determine the money generated by the UK from domestic seafood production and exports *vs.* spent on imports, the annual value of domestic landings, imports, and exports was digitised using UK Sea Fisheries Statistics tables (Table S1). The value of UK-based aquaculture production was sourced from FAO (FAO 2021b; Table S1). All values were adjusted for inflation based on the Retail Prices Index (ONS 2022), adjusted to the year 2000 as is the standard in recent MMO reports.

### Species composition

To analyse changes in the relative abundance of the finfish and shellfish ‘species’ produced and traded by the UK through time, we identified the most commonly traded seafood taxa today (based on average imports and exports over the last 15 years; 2006–2020). These seven species or taxonomic groups (herein referred to only as ‘species’; Atlantic cod (*Gadus morhua*), haddock (*Melanogrammus aeglefinus*), Atlantic herring (*Clupea harengus*), Atlantic mackerel (*Scomber scombrus*), salmon (various spp.), tuna (various spp.), and shrimps/prawns (various spp.)) represent the majority of seafood produced (57.8% ± SE 1.2% (n = 14)), imported ﻿(64﻿.1% ± SE 0.5% (n = 15)) and exported (59.2% ± SE 1.1% (n = 15)) during this period. The annual weight of seafood produced by the UK, binned into these seven species and ‘Other finfish’ and ‘Other shellfish’ was obtained from ICES catch statistics and FAO aquaculture production datasets respectively ﻿(FAO 2021b; ICES﻿ 2020). Note that ICES catch data were used in this particular analysis as shellfish landings data were not available in weight between 1900 and 1961 in the UK Sea Fisheries Statistics tables. Import and export weights were digitised for the same taxonomic groups from the UK Sea Fisheries Statistics tables (Table S1) for each year between 1965 and 2020, and at least every five years between 1900 and 1964. Import and export weights were digitised more frequently than every five years during the two world wars to capture short-term changes in trade during these pivotal periods. Imports and export weights are often separated by good type in the data source (e.g., salted *vs.* frozen), which were summed to produce annual total weights for each species.

To explore the mismatches between the seafood produced vs. consumed in the UK at the species level, historical trends in production and trade were analysed for six of the species highlighted above (tuna were excluded as UK landings and aquaculture production has always been negligible). The annual weight of seafood produced, imported, and exported by the UK was obtained from the UK Sea Fisheries Statistics tables and FAO aquaculture production datasets (FAO 2021b; Table S1).

### Influence of policy change on fish production and consumption

Mismatches between the seafood produced *vs.* consumed in the UK were explored by using the total weight of seafood produced, available for consumption and traded annually to calculate an annual ‘import mismatch’ (imported/consumed) and ‘export mismatch’ (exported/produced) metric, using processed weights for production and consumption estimates (see above). 

Generalised additive models (GAMs) were fitted to annual mismatch estimates (‘import mismatch’ (imported/consumed) and ‘export mismatch’ (exported/produced)) using the restricted maximum likelihood approach to explore changes through time. GAMs were fitted using the *mgcv* package (version 1.8.35; Wood 2017) in R (version 4.1.0; R Core Team 2021). Model diagnostics were checked using the gam.check() function in the *mgcv* package and the *DHARMa* (version 0.4.3; Hartig 2021) package. The basis dimension of each model was increased incrementally to improve model fit using the model diagnostics, as described in Simpson (2018). We then used the *segmented* package (version 1.3.4; Muggeo 2017) to ascertain whether breakpoints existed within each of the time series, and whether they coincided with major policy changes. The selgmented() function was used to select the optimal number of breakpoints based on Bayesian information criterion.

To further explore whether mismatches between UK fish production and consumption were influenced by major political events or policy changes, we identified policy states that were likely to have affected UK fishing and trade over the last 120 years from previous publications (Table 1). Two Generalized Linear Models (GLMs) were fitted using the import mismatch and export mismatch as the response variables, and year and policy state as the predictor variables. Data were log-transformed when necessary to meet assumptions of normality and homogeneity of variance. Tukey tests were used to test for differences in mismatch extent between periods using the glht() function in the *multcomp* package (version 1.4.17; Hothorn et al. 2021).

**Table 1:** Regulatory landscapes that are likely to have influenced UK landings and trade between 1900 and 2022 and their corresponding years

Years	Regulatory landscape	Policy state	Description
1900–1913	Open access	Open access	Thomas Huxley stated in 1883: "Probably all the great sea fisheries are inexhaustible; that is to say, that nothing we do seriously affects the numbers of fish. Any attempt to regulate these fisheries seems consequently, from the nature of the case, to be useless" (Huxley 1883). This quotation summarises UK fisheries policy during the period, which was largely unrestricted following the Sea Fishing Act of 1868, in which Huxley was a commissioner (Robinson 1997). It is worth noting, however, that there was concern about the overexploitation of fish populations during this period, which instigated the creation of the International Council for the Exploration of the Sea (ICES) and the Marine Biological Association’s laboratory in Lowestoft in 1902 (Cefas 2020; Went 1972). There were also some government enquiries and studies which showed declines in catch per unit effort (Poulsen & Holm 2007; Robinson 1997). Fish and chip shops increased in popularity, and it is estimated that 20% of white flaky fish, e.g., cod and haddock, landed by Great Britain was used by fish and chip shops (Franklin 1997; Kerby et al. 2012)
1914–1918	World War I	World Wars	The world wars largely affected UK fisheries: Access to many fishing waters was restricted to avoid enemy attacks, fishing vessels were used by the navy, and fishers were enlisted for military service (Kerby et al. 2012)
1919–1938	Open access	Open access	There was an expansion of distant-water fisheries during the inter-war years, particularly in the Arctic and West Africa, as the use of steam-powered trawlers increased (Thurstan et al. 2010). Fishing remained largely unrestricted during the period (Kerby et al. 2012). Fish and chip shops continued to increase in popularity, and it is estimated that 60% of white flaky fish landed by Great Britain was used by fish and chip shops (Kerby et al. 2012)
1939–1945	World War II	World Wars	See "World War I"
1946–1975	Open access	Open access	Improved technology allowed UK fisheries to recover following World War II, as the UK returned to its previous fishing grounds, and fishing policy remained largely the same for much of the period (Holm 2012). However, fishing restrictions started to change following the Cod Wars (1958–61, 1972–73, and 1975–76), which were three disputes over fishing rights between the UK and Iceland (Engelhard 2005). This period came to an end following the third Cod War when Iceland introduced an Exclusive Economic Zone (EEZ) of 200 nautical miles, which many other countries had adopted by the mid-1970s (Kerby et al. 2012). UK consumption of seafood declined during this period, likely due to increased prices, lack of availability, and competition from other food types (Kerby et al. 2012; Thurstan & Roberts 2014). Additionally, a stock collapse of herring and consequent total fishing ban resulted in declined popularity for kippers, even after the herring stock recovered (Dickey-Collas et al. 2010)
1976–2020	EEZ/EU	EEZ/EU	The widespread adoption of EEZs resulted in the decline of UK distant-water fisheries, including those in Iceland, the Barents Sea, Greenland, and Norway (Kerby et al. 2012). Moreover in 1973, the UK joined the European Economic Community (EEC), which later became part of the European Union (EU) (Treaty on European Union 1992). The EEC imposed fishing restrictions through the Common Fisheries Policy (CFP), developed between 1976 and 1983, including a Total Allowable Catch for species and quotas for member states (Symes 1997). The UK joining the EEC also included joining the European Single Market, which removed many of the trade barriers between member states (Phillipson and Symes 2018). Consumer demand for cod and haddock remained relatively high, despite the decline in distant-water fisheries causing reduced landings of these species (Franklin 1997; Kerby et al. 2012). There were increased consumer preferences for new types of fish not landed in the UK, including tuna and prawns (Harmsen and Traill 1997; Jaffrey and Brown 2008). In 2020, the COVID-19 pandemic impacted global fisheries and aquaculture production, seafood trade and consumption (FAO 2022). This included a decline in capture fisheries production and reduced international trade due to lockdowns and border restrictions (FAO 2022). As COVID-19 only impacted seafood production and trade in one year of the 120-year analysis, we did not include COVID-19 formally as a factor in the analysis
2021-Present	Brexit	Not included in model due to lack of trade data	The UK withdrew from the EU in December 2020, and consequently the CFP, resulting in the UK having control of fishing regulations in the UK EEZ (Phillipson and Symes 2018; Stewart et al. 2022). However, as Brexit also includes the UK leaving the European Single Market (Cabinet Office 2021), it may result in greater trade barriers, including new tariffs on fish caught outside of the UK EEZ and custom controls, which affect fish imports and exports (no data available at time of writing) (Seafish 2021). The big five species remain the most consumed seafood in the UK: cod, haddock, tuna, salmon and prawns (MMO 2021b)

Three key policy states were identified across the study period: (1) Open access policy, (2) World Wars, and (3) Exclusive Economic Zone (EEZ) and European Union (EU) policy. Although mentioned in this table, trade data were not available for the post-Brexit period, so this period was not included in the analysis

## Results

### Seafood production and trade over time

**Fig. 1:**
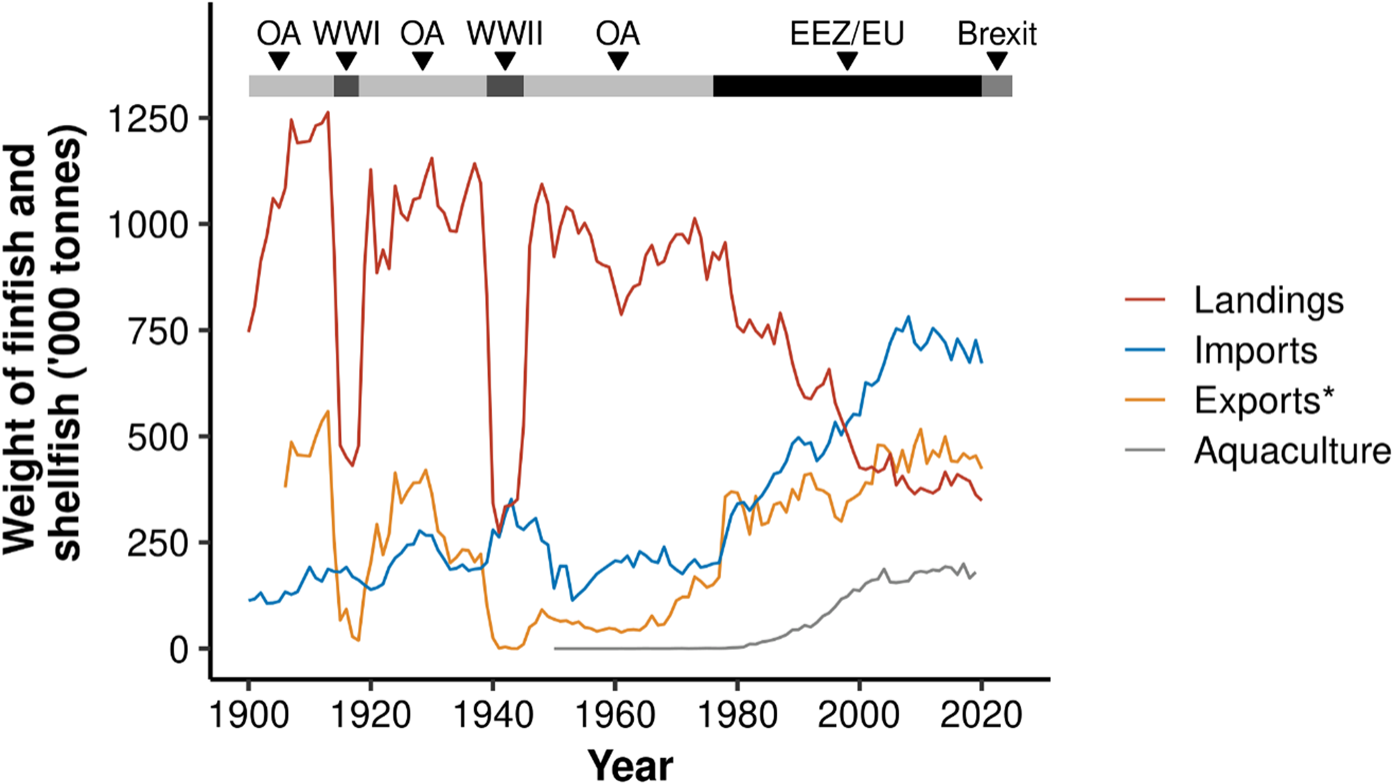
Changes in UK finfish and shellfish domestic landings (red), imports (blue) and exports (yellow), and aquaculture production (grey) between 1900 and 2020. Note that landings and aquaculture represent pre-processed weights, while most imports and exports are processed. Note that export weights (*) are not independent of the other datasets, as UK export data will include some domestic landings, aquaculture and re-imports, as well as UK vessel landings abroad. Major periods are shown above plot (OA = Open access policy, WW = World wars, EEZ/EU = Exclusive Economic Zone and European Union policy)

UK seafood production and trade have gone through major changes over the last 120 years (Fig. 1). In the first half of the twentieth century, the UK was consistently landing large quantities of wild captured fish and shellfish, with landings peaking in 1913 at 1.26 million tonnes. During the two world wars, landings dropped by more than half, but rebounded quickly following each event. Total exports showed similar trends during this period: the UK was exporting large quantities of seafood before World War II, but large declines were observed during both world wars. However, following World War II, exports did not return to previous levels and remained relatively low until the 1970s, when they started to increase. Following the introduction of EEZs (hence exclusion from Icelandic waters) and the UK joining the EU in the mid-1970s, UK domestic landings declined rapidly, from 869 thousand tonnes in 1975 to 349 thousand tonnes in 2020. In parallel, there has been a rapid increase in seafood imports, which were relatively low before the 1970s (Fig. 1). UK-based aquaculture production also increased considerably in the 1980s and 1990s, peaking at 34% of domestic seafood production in 2012.

### Seafood consumption

**Fig. 2:**
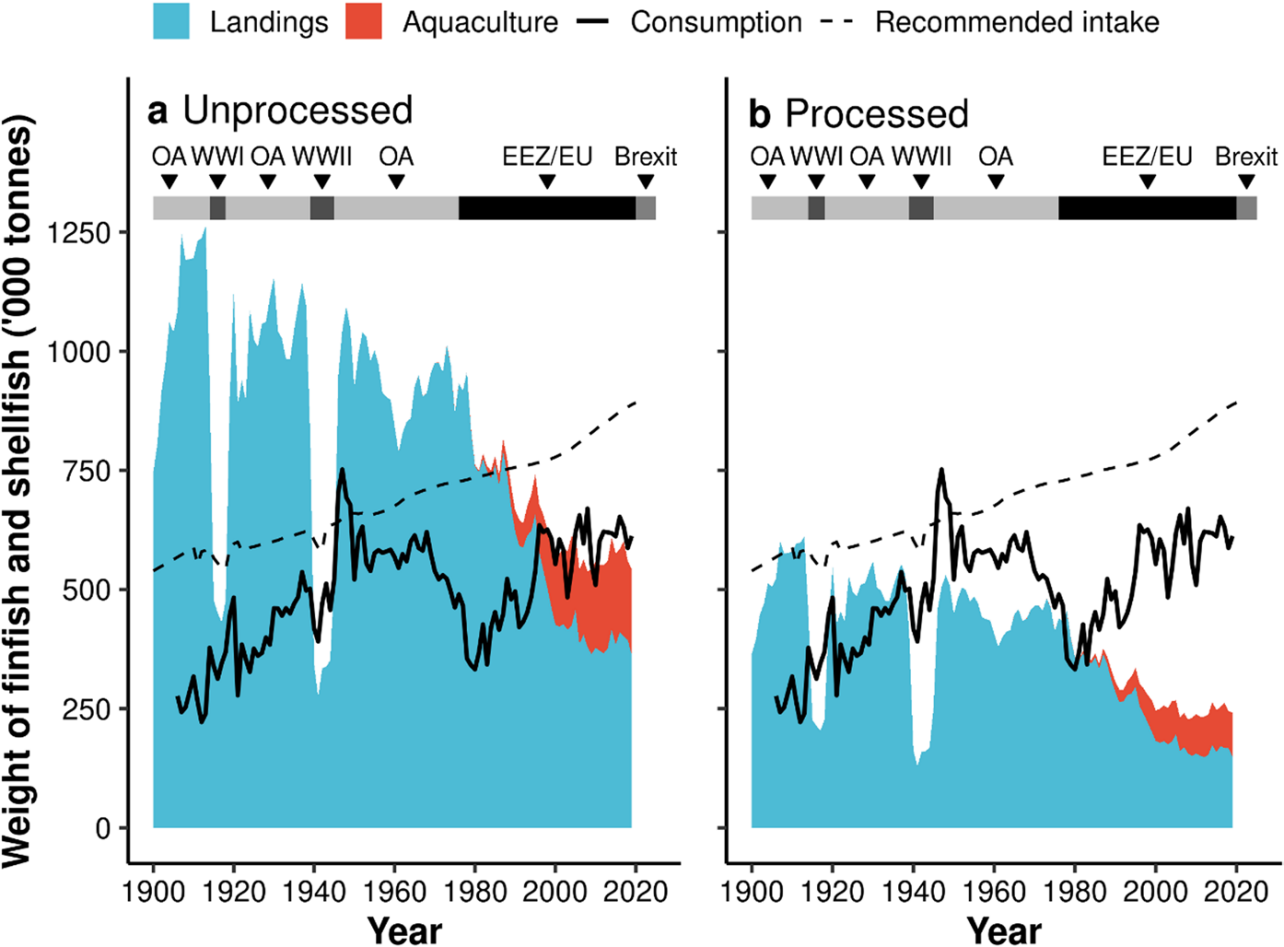
Changes in UK domestic landings (blue filled area) and aquaculture production (red filled area) contrasted with observed (solid line) vs. recommended (dashed line) fish intake levels by the UK public between 1900 and 2019. Plots show landings and aquaculture before (**a**) and after (**b**) processing. Consumption was estimated as the weight of processed landings (in the UK and abroad) and aquaculture plus imports minus exports. Recommended intake represents the Food Standards Agency’s fish recommendations for the UK adjusted to population size. Major periods are shown at the top (OA = Open access policy, WW = World wars, EEZ/EU = Exclusive Economic Zone and European Union policy)

Over most of the last 120 years, and particularly over the last 50 years, UK seafood production (processed landings and aquaculture) has been unable to meet the observed or the recommended annual intake of fish (Fig. 2b). Prior to both wars, processed landings could have provided all of the seafood consumed in the UK, with a surplus, allowing the UK to be a significant exporter (Fig. 2b). However, following the introduction of EEZs and EU policy in the mid-1970s, domestic seafood production has been increasingly unable to meet consumer demand, with the proportion of seafood consumed that could have been provided through domestic production dropping from 89% in 1975 to 40% in 2019.

When UK landings peaked in 1913, 100% of the recommended fish intake could have been attained from processed domestic landings alone, although actual fish consumption was considerably lower, with the UK exporting much of the seafood landed during this period. Following World War II, UK fish consumption briefly surpassed the recommended intake, but landings alone could no longer support consumer demand nor supply the recommended intake levels. Since the adoption of EEZs and EU policies, the gap between domestic fish production and the recommended intake has continued to widen, with the proportion of the recommended intake available from locally produced fish dropping from 57% in 1975 to 27% in 2019.

While purely a hypothetical scenario, domestic production alone could almost entirely meet demand if seafood were consumed before processing i.e., the entire animal (apart from the guts of finfish) (Fig. 2a). The gap between domestic production and recommended fish intake, which is higher than current demand, would also decrease by 34%, however there would still be a 39% deficit based on the current population size (Fig. 2a).

### Economics

**Fig. 3:**
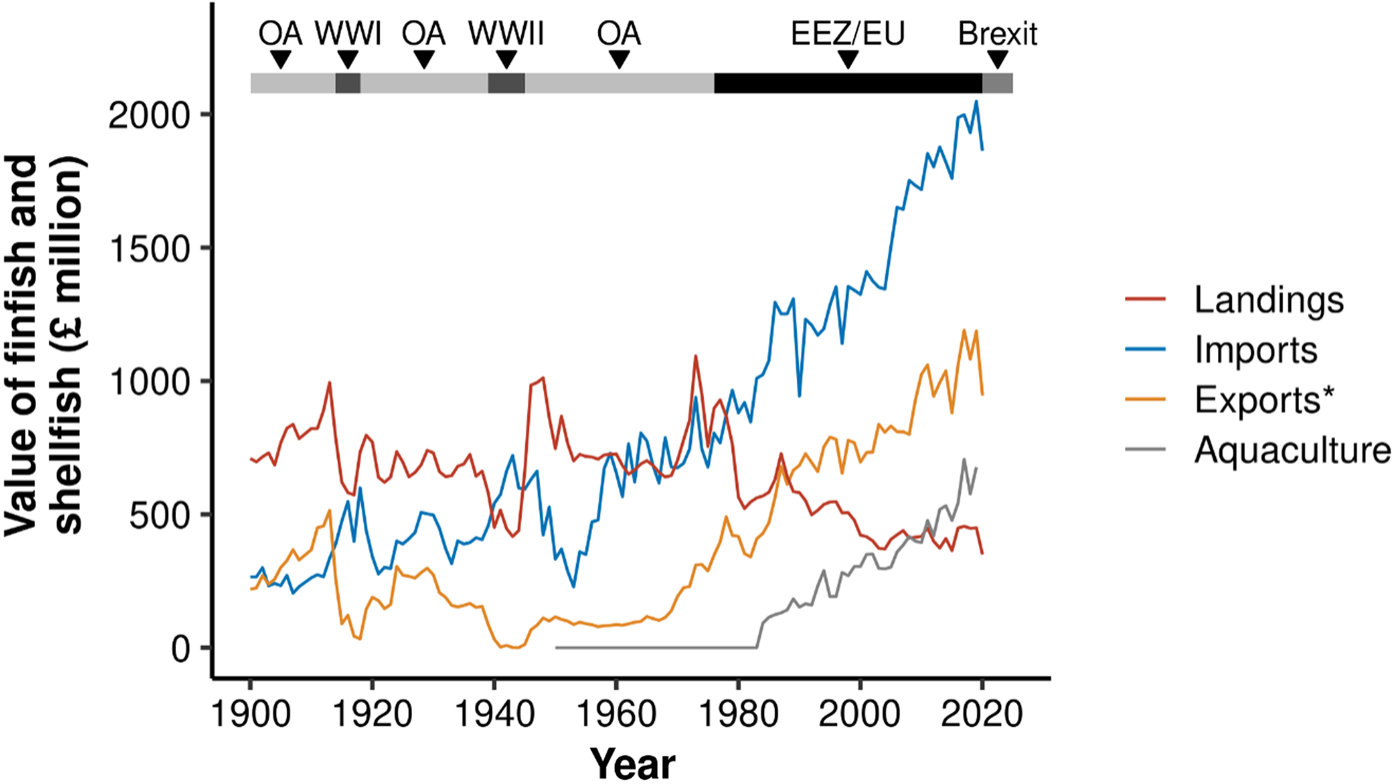
Trends in the value of UK finfish and shellfish domestic landings at point of landing (red), imports (blue), exports (yellow), and aquaculture production (grey) between 1900 and 2020. Value is adjusted for inflation based on the Retail Prices Index with the year 2000 as the base year. Note that the value of exports (*) is not independent of the other datasets, as UK export data will include some domestic landings, aquaculture and re-imports, as well as UK vessel landings abroad. Major periods are shown at the top (OA = Open access policy, WW = World wars, EEZ/EU = Exclusive Economic Zone and European Union policy)

The widening gap between consumer demand and domestic production has economic implications, as the UK is spending increasingly more on imports to meet seafood requirements (Fig. 3). Before the mid-1970s, the total value of UK landings was high, with the highest value (at the point of landing) in 1973 at £1094 million after adjusting for inflation. Following the introduction of EEZ and EU policies, the money generated from UK fleet landings declined dramatically (Fig. 3). This is due to a combination of reduced landings and catch composition changes towards lower economic value seafood species (e.g., mackerel, herring) with low UK consumer demand. However, the rise in UK-based aquaculture has masked the loss of value from landings, and the total value of domestic fish production has remained relatively stable over the last 120 years.

The UK’s spending on imports has increased dramatically, particularly since the introduction of EEZs and EU policy. Between 1975 and 2020, outgoings on imports rose by 175% from £0.7 billion to more than £1.8 billion (Fig. 3). This represents an increase from £12 per capita in 1975 to £28 per capita in 2020, after adjusting for inflation. There has also been a considerable rise in the value of exports, which has increased in parallel with the value of domestic aquaculture production. Despite this increased income from exports, there has been a notable rise in the UK’s trade deficit (imports value minus exports value), which has grown from £389 million in 1975 to £917 million in 2020. It is worth noting that the rise in the UK’s trade deficit is positive from the public health perspective as the available quantity of seafood becomes closer to the recommended intake.

### Species composition

#### Seafood production

**Fig. 4:**
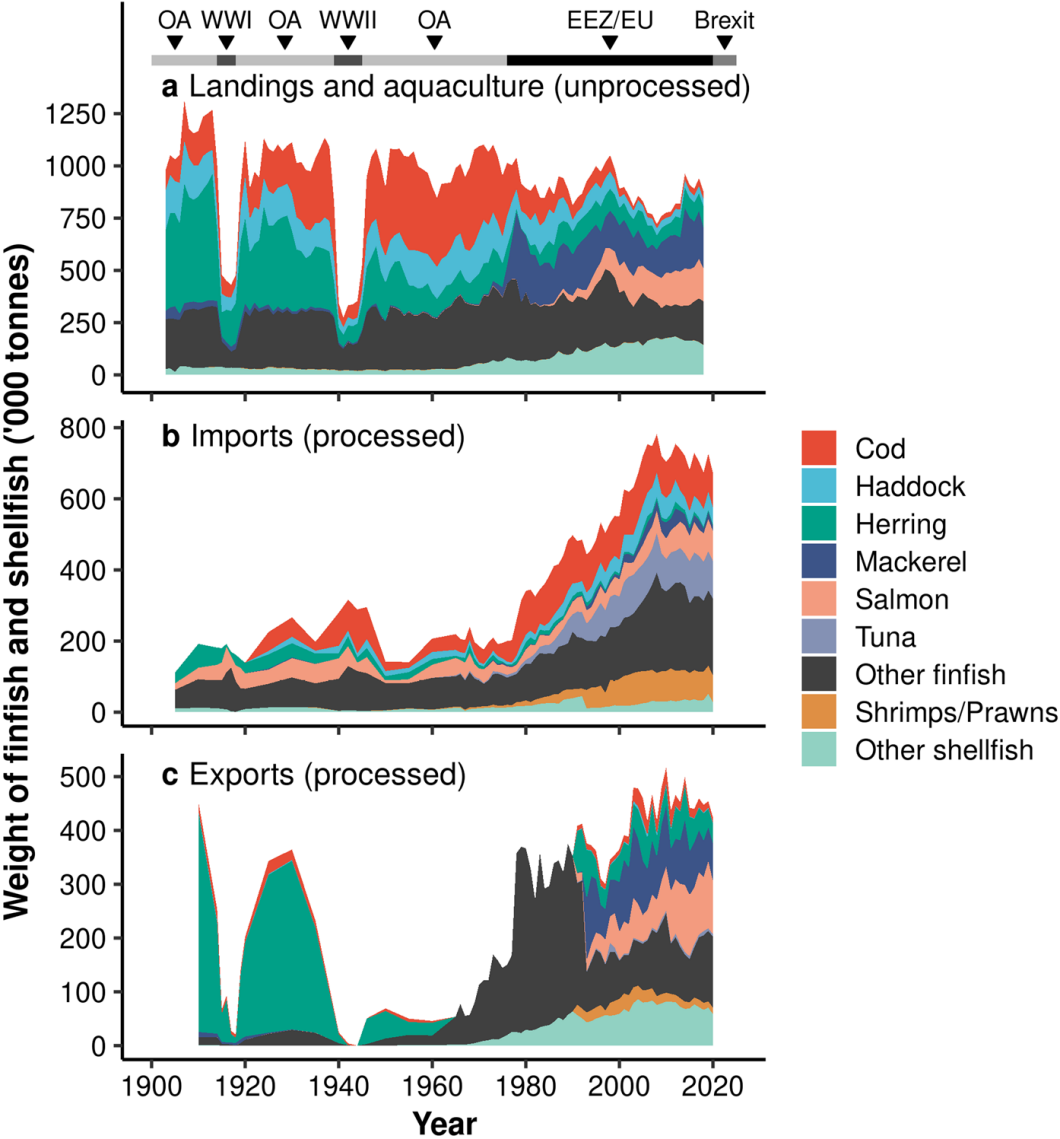
Changes in the species composition of UK finfish and shellfish production (**a**), imports (**b**) and exports (**c**) between 1903 and 2020. Total landings and aquaculture production (unprocessed) by the UK are shown, whereas most imported and exported seafood products are processed. Exports data to the species level was not available between 1965 and 1990, so “Other finfish” and “Other shellfish” represent all finfish and shellfish during this period, but otherwise representing all fish species not specified. Major periods are shown at the top (OA = Open access policy, WW = World wars, EEZ/EU = Exclusive Economic Zone and European Union policy)

There have been major changes in the species composition of seafood produced by the UK over the last century (Fig. 4a). From the start of the twentieth century to the mid-1970s, cod, herring, and haddock dominated landings (mean = 67.0% ± SE 0.6% of total landings weight, n = 73 years). However, following the introduction of EEZs and EU policies, there were large increases in the landings of mackerel and shellfish species, particularly crabs, scallops, and *Nephrops*, and large decreases in herring and cod landings (Fig. 4a).

The rapid increase in the UK aquaculture industry in the 1980s also affected the composition of domestic seafood production in recent years. UK-based aquaculture is dominated by relatively few groups, namely Atlantic salmon (*Salmo salar*), shellfish and rainbow trout (*Oncorhynchus mykiss*) (86.8%, 7.5% and 5.4% of total aquaculture production weight in 2019, respectively). As such, there has been a large increase in the overall contribution of salmon, which are rarely landed in the UK, and shellfish (Fig. 4a). Between 1976 and 2018, mackerel, salmon, and shellfish together accounted for an average of 46.9% ± 1.9% SE (n = 43) of total seafood production, compared to only 25.7% ± 1.3% SE (n = 43) for cod, haddock, and herring, the historically most important wild-capture species combined.

#### Trade

**Fig. 5:**
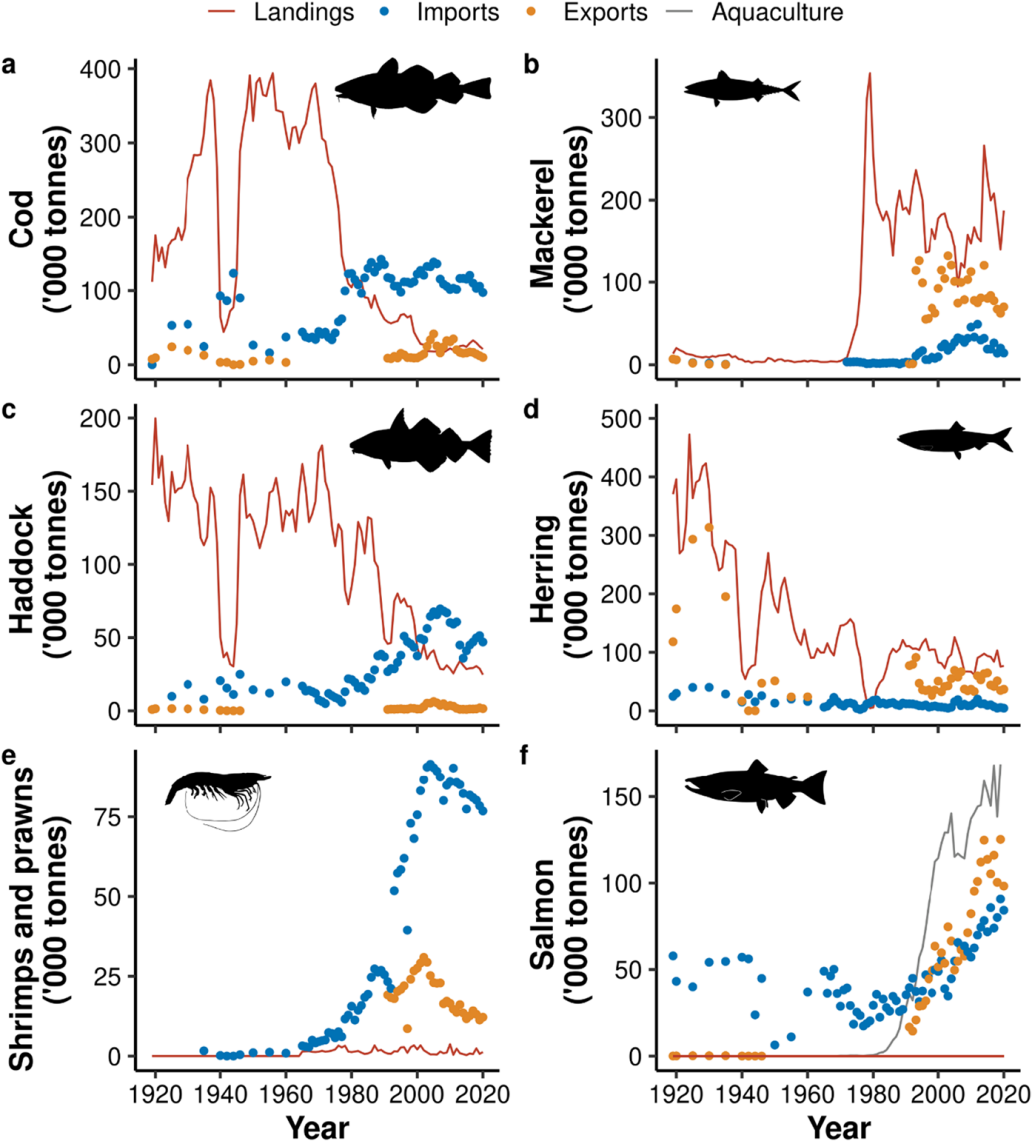
Changes in UK landings (red), imports (blue), exports (yellow), and aquaculture production (grey) of (**a**) Atlantic cod (*Gadus morhua*), (**b**) haddock (*Melanogrammus aeglefinus*), (**c**) Atlantic herring (*Clupea harengus*), (**d**) Atlantic mackerel (*Scomber scombrus*), (**e**) shrimps and prawns (various species), and (**f**) salmon (various species) between 1919 and 2020. Annual landings data for each species was not available before 1919. UK-based aquaculture production and UK vessel landings in the UK and abroad are shown

Not only has there been a substantial increase in the total quantity of imports and exports (Fig. 1), the trading rates of different species have also changed through time (Fig. 4). Today, the most imported taxa are cod, tuna, shrimps and prawns, salmon, and haddock (Table 2). Cod and haddock have been consistently important import species, but following the landing restrictions imposed by EEZs and EU policies in the 1970s, their import rates rose rapidly to meet ongoing demand and constrained fishing opportunities for the UK fleet (Fig. 5a and c). In recent years, there have also been large increases in imports of tuna and shellfish species (Figs. 4 and 5), the latter dominated by shrimps and prawns (> 74% in 2020), over crabs, lobsters or molluscs.

**Table 2:** The UK’s currently most traded seafood taxa, shown as the proportions (by weight) of total UK fish imports and exports consisting of selected species or species groups between 2006 and 2020

Species	Proportion of imports between 2006 and 2020 (%)	Proportion of exports between 2006 and 2020 (%)	Trade imbalance
Cod	**15.30 ± 0.32**	**4.44 ± 0.43**	I > E
Tuna	**14.46 ± 0.44**	1.11 ± 0.08	I > E
Shrimps/Prawns	**11.53 ± 0.14**	**3.37 ± 0.22**	I > E
Haddock	**7.48 ± 0.34**	0.53 ± 0.09	I > E
Salmon	**10.02 ± 0.42**	**20.83 ± 1.32**	E > I
Mackerel	3.91 ± 0.35	**18.45 ± 0.72**	E > I
Herring	1.41 ± 0.15	**10.51 ± 0.70**	E > I
Other fish	35.89 ± 0.50	40.75 ± 1.09	E > I

Mean proportions ± standard errors are shown (n = 15 years). The five species with the highest mean proportions of imports and exports (shown in bold) were selected. “Trade imbalance” shows whether the mean proportion of imports (I) or exports (E) of each group is greater. “Other fish” represents all fish species not specified

Across the 120-year time series, exports from the UK have been dominated by herring and mackerel, suggesting a relatively low national demand (Figs. 4 and 5). Herring dominated exports in the first half of the twentieth century, accounting for an average of 69.9% ± 5.4% SE (n = 18) of total exports in 1900–1964, and 85.4% ± 0.4% SE (n = 5) between the world wars (Figs. 4c and 5d). Mackerel landings were relatively low until the mid-1970s, after which they increased rapidly from 48 thousand tonnes in 1975 to a peak of 353 thousand tonnes in 1979 (Fig. 5b). Exports data were not available to species level between 1965 and 1990, but it is evident that there was a large shift in the species composition during this period. At least from 1991, herring no longer dominated the export market, and there was a higher diversity in the species exported, with mackerel, salmon, and shellfish species, such as crabs and *Nephrops*, being exported in increasing quantities (Fig. 4c).

Seafood imports are typically dominated by species with high consumer demand but limited domestic production such as cod or shrimps and prawns (Fig. 5e). However, both imports and exports of salmon products have increased alongside the rapid growth of the UK salmon aquaculture industry (Fig. 5f), suggesting that much of the salmon produced domestically is exported. Between 2006 and 2020, the mean annual value per kg of UK salmon imports and exports (MMO 2014, 2021a), after adjusting for inflation, were £3.31 ± SE £0.14 (n = 15) and £3.42 ± SE £0.10 (n = 15), respectively, suggesting there is not a large difference between the value of UK salmon imports and exports.

### Influence of policy change on fish production and consumption

**Fig. 6:**
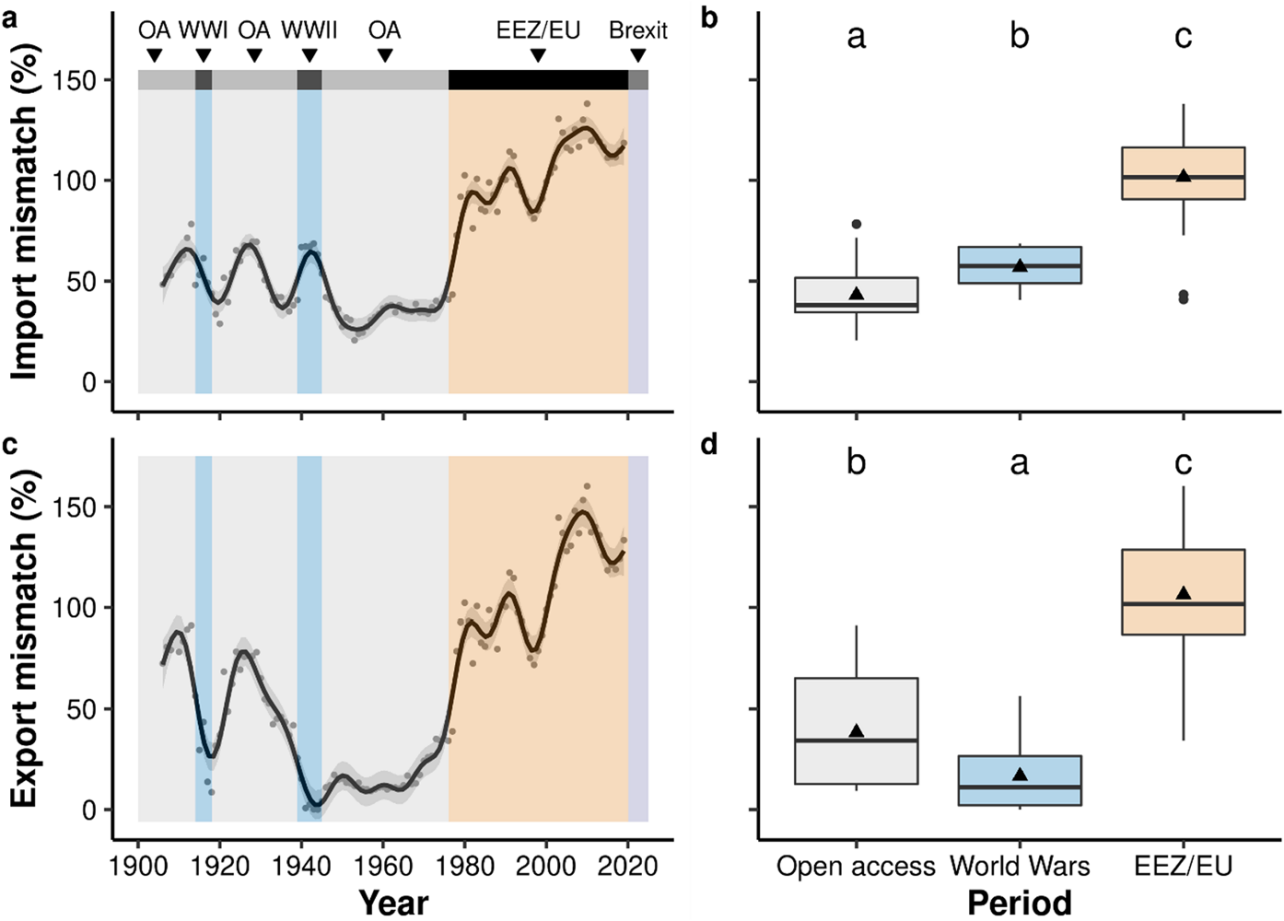
Changes in the fraction of seafood consumed in the UK that is imported [‘import mismatch’] (**a**) and the fraction of UK seafood production that is exported [‘export mismatch’] (**c**) between 1906 and 2019. Black trend line and bounding grey area represent fitted GAM smooths ± 95% confidence intervals. Major periods are shown at the top and by shaded boxes (OA = Open access policy (grey), WW = World wars (blue), EEZ/EU = Exclusive Economic Zone and European Union policy (orange)). Boxplots show differences in median import (**b**) and export (**d**) mismatches between policy periods. Bold lines show median values, whiskers show maxima and minima, and triangles show mean values. Letters indicate statistically significant differences from Tukey tests (*p* < 0.05). Import and export mismatches exceed 100% in some years, likely due to re-imports, re-exports, and a relatively coarse approach to convert landings weight into processed weight

Since the introduction of EEZs and EU policy, the UK has increasingly imported the majority of seafood consumed (average of 102% ± SE 3% (n = 44) in 1976–2019) and exported the majority of seafood it produces from landings and aquaculture (average of 106% ± SE 4% (n = 44) in 1976–2019). Note that both estimates are slightly higher than 100%, likely due to a combination of re-imports, re-exports, and a relatively coarse approach to convert landings weight into processed weight (see Methods). This has resulted in large increases in import (Fig. 6a) and export (Fig. 6c) mismatches in recent decades. Both metrics showed significant, non-linear trends over time, with sharp increases in the mid-1970s (Fig. 6a; GAM; Deviance explained = 96.8%, edf = 21.9, *F*_24_ = 116.1, *p* < 0.001 and Fig. 6c; GAM; Deviance explained = 97.2%, edf = 21.8, *F*_23_ = 130.3, *p* < 0.001, respectively). Breakpoints in the import mismatch time series coincided exactly with the introduction of EEZs (exclusion of UK fleets from Icelandic waters) and EU policies (Fig. 6a; Segmented regression; Estimated breakpoints = 1975, 1979, *p* < 0.001). Conversely, the first breakpoint in the export mismatch time series was about 15 years earlier, during a period of open access but shortly after the first cod war (1958–1961) started (Fig. 6c; Estimated breakpoints = 1959, 2010, *p* < 0.001). The import and export mismatches were significantly greater during the EEZ and EU period than during the world wars and open access periods, suggesting that the introduction of EEZs and the UK joining the EU altered patterns in imports and exports significantly more than the world wars (Tukey tests, *p* < 0.05; Fig. 6b and d). Additionally, the import mismatch was higher during the world wars than the open access periods, whilst the export mismatch was lower (Tukey tests, *p* < 0.05; Fig. 6b and d).

## Discussion

This study highlights how UK landings and aquaculture production are increasingly unable to meet consumer demands, in terms of either total weight or preferred species. At the beginning of the twentieth century, the UK was consistently landing large quantities of wild captured fish and shellfish, allowing the UK to be a net exporter of seafood. The world wars created a mismatch between seafood production and consumption, as the UK relied on imports to meet demand as domestic fishing declined; however, the fishing industry recovered quickly following both world wars (Kerby et al. 2012). Today, the UK is a net-importer of seafood, with the mismatches between production and consumption becoming increasingly evident after the introduction of EEZs and the UK joining the EU in the mid-1970s, which had a greater impact upon trade and domestic production patterns than either of the world wars. The driving factor appears to be strong consumer preference for large, flaky fishes, particularly cod and haddock, from when the UK had a thriving distant-water fishery (Jennings et al. 2016). Today these species are landed in low quantities in UK waters, while small, bony species, particularly mackerel and herring, are landed in high quantities and primarily exported to the Netherlands and France (Jennings et al. 2016; MMO 2021b). The mismatch indicated by high local production and limited local demand for these cheap and nutritious species is probably due to consumer aversion to oily and bony fish (Franklin 1997; Leek et al. 2000). Herring was landed and exported in high quantities across the time-series, suggesting that consumer demand for herring has been low since the 1900s. Similar trends have been observed in Ireland, where consumer preferences for seafood have not adapted to the fish locally available, creating mismatches between seafood landings, and imports and exports (Miller et al. 2012). There are clear economic implications, as the UK is spending increasingly more on imports than it is generating from exports. Importantly, the economic consequences of a changing seafood industry have resulted in considerable variations at a regional level, with the loss of the distant water fleets particularly impacting coastal communities in East England (Kerby et al. 2012), and the rapid rise in salmon aquaculture creating jobs and economic gains in many rural areas of Scotland (Callaway et al. 2012; Graziano et al. 2018; Uberoi et al. 2022). The mismatch between domestic production and consumption also has health implications, as the UK is currently not producing sufficient seafood to meet recommended intake levels. This finding aligns with Thurstan and Roberts (2014) who found that UK domestic landings were 81% below recommended levels in 2012. The present study shows that this gap has widened further, with landings in 2019 being 83% below recommended levels. Even when aquaculture production is included, total domestic UK seafood production is still 73% lower than recommended levels (2019 recommended intake vs. total production = 887.92 vs. 241.93 thousand tonnes, respectively; Fig. 2b)﻿﻿.

### Changes in UK seafood production, trade, and consumption

During the last 120 years, UK fisheries production and trade have been subject to major changes driven by policy shifts, geo-political upheavals, and technological innovation, among other factors. Perhaps unsurprisingly, change points in production and trade patterns were observed during the world wars and as the first of the ‘cod wars’ unfolded in the mid-twentieth century. It is also unsurprising that the expansion of EEZs and the UK’s incorporation into the EU, which both occurred during the mid-1970s, also created a shift in UK fish landings and trade. However, these later policy shifts resulted in large-scale import and export mismatches that had never been previously observed. Given that the most seismic shift in UK fish production and trade occurred at this point, this presents a concerning picture for the UK’s recent move out of the EU which may potentially alter the fish available to the UK fleet from the EU EEZ, which is to be negotiated annually from 2026 (Trade and Cooperation Agreement 2020).

The mismatches observed during the world wars occurred primarily due to large drops in UK landings due to fishing waters being closed, fishing vessels being deployed by the navy, and fishers being enlisted for military service (Kerby et al. 2012). Aside from these periods, landings were consistently high in the first half of the twentieth century due to the heavy reliance upon distant-water fisheries. The expansion of distant-water fisheries was accelerated by the increased popularity of fish and chip shops, which resulted in a rapid rise in seafood consumption until the early 1950s (Kerby et al. 2012).

UK seafood consumption decreased in the 1950s, likely due to increased price of fish and competition from other food sources (Thurstan and Roberts 2014). Additionally, landings declined rapidly from the mid-1970s with the introduction of EEZs around countries’ coastlines previously fished by UK distant-water fisheries, and the UK joining the CFP, which imposed reduced fishing quotas in the North Sea. This had implications for consumers, as the UK could no longer supply consumer demand for popular seafood types, such as cod and haddock (Kerby et al. 2012). This may explain the large increase in imported fish to supplement consumer demand, alongside the popularisation of alternative foods such as the battered sausage, which was invented in the 1970s due to the decline in cod availability (National Federation of Fish Friers 2019).

Policy and fish stock changes also affected the species composition of the UK fish supply. Eastern Bloc vessels were fishing a large mackerel stock in European waters from the 1960s, which they were no longer allowed to do following the introduction of EEZs (Whitmarsh and Young 1985). As the UK had excess vessels that were previously used for distant-water fisheries, they took advantage of this large stock, and mackerel landings increased dramatically (Whitmarsh and Young 1985). Additionally, the herring stock in the North Sea collapsed almost entirely during the 1970s, resulting in a large decline in UK herring landings (Dickey-Collas et al. 2010). There was a total ban on herring fishing in the UK EEZ, which resulted in the closure of many fisheries (Dickey-Collas et al. 2010). The ban also caused a shift in consumer preferences, as demand for kippers declined following the herring ban (Dickey-Collas et al. 2010). The ban on herring fishing allowed the stock to recover in the 1980s, but even with increased availability, local demand for herring remained low and the UK shifted its focus to herring exports (Dickey-Collas et al. 2010).

The UK joining the European Single Market in 1973 removed the trade barriers between the UK and European countries which increased the ease of international trade (Phillipson and Symes 2018). Although exports had been high at the beginning of the twentieth century, exports had not recovered following their decline in World War II. The new trade deal with European states revitalised UK exports, particularly in mackerel and shellfish which were now landed in high quantities despite low UK consumer demand (Graziano et al. 2018). Additionally, as Scottish salmon aquaculture production increased in the 1980s, the trade deal allowed large quantities of salmon to be exported to European countries, such as France, Ireland, and Poland (Graziano et al. 2018).

The UK joining the European Single Market in 1973, and increased globalisation enabling trade with countries outside of the EU, influenced seafood imports, which increased considerably from the mid-1970s. Imports in traditionally consumed species, such as cod and haddock, rose rapidly to supplement decreased landings, alongside increased imports of untraditional foods, namely tuna, shrimps, and prawns. As more fish were now available for the UK, seafood consumption began to rise, although the taxonomic composition of the seafood consumed had changed from earlier in the twentieth century (Thurstan and Roberts 2014). The increased ease of international trade, the changes in consumer preferences, and the decreased availability of fish from landings have resulted in UK consumers increasingly relying on imports to meet seafood requirements.

### Implications for UK food security

Jennings et al. (2016) defined food security as a seafood supply that is “sufficient, safe, sustainable, shockproof, and sound”, and concluded that the UK seafood supply was secure, as consumer demand could be attained by high levels of imports. However, this makes the assumption that trade pathways are reliable and secure, which has been brought sharply into question since the Brexit referendum.

Our findings suggest that consumer preference is a major driver of which seafood products are imported and exported, in support of the conclusions of Jennings et al. (2016). While Jennings et al. (2016) focused on seafood trade in 1995–2014, our longer-term analyses revealed that the mismatch between production and consumption exhibited a step-change following the introduction of EEZs and EU policy, greater even than the changes resulting from the world wars. Despite increases in the popularity of tuna, shrimps, and prawns, UK consumer preferences have largely remained consistent through time, and have not responded to changes in local production, which is particularly evident for cod and haddock. Distribution shifts due to climate change have further reduced the UK’s availability of these cold-water species, which are largely imported from countries north of the UK such as Norway and Iceland (Jennings et al. 2016; Phillipson and Symes 2018; Pinnegar et al. 2017). Despite thriving herring and mackerel fisheries, there is limited local demand for these cheap and nutritious species, probably due to consumer aversion to oily and bony fish (Franklin 1997; Leek et al. 2000), resulting in the UK exporting the majority of these landings.

Brexit will change the trade and fishing agreements between the UK and EU countries, creating uncertainty around the availability and cost of seafood (Phillipson and Symes 2018). A similar change in seafood availability was observed following the introduction of EEZs and the CFP in the mid-1970s, where seafood types in high demand were no longer locally available (Fig. 4). A key difference in the UK’s current situation is that the UK no longer has recourse for unrestricted EU imports to supply demand (Seafish 2021). The UK exports approximately half of the fish that it lands to EU countries, representing two-thirds of total exports, and imports a third of its total fish imports from the EU (Phillipson and Symes 2018). Additionally, trade agreements with other nations significant for UK fisheries, namely Norway, have also been affected by Brexit (Bjørndal and Munro 2021). Already within the first year of Brexit, there have been numerous reports on its effect on UK fisheries. For example, in the first few months, seafood was held at EU borders due to new trade regulations (Dickins 2021), a large UK trawler destined for Norway was grounded due to the lack of a fisheries agreement (BBC News 2021a), and trade restrictions were placed on UK fishers exporting shellfish to the EU (BBC News 2021b). As the final Brexit deal also coincided with the COVID-19 pandemic, the most recent changes in landings and trade will be partly explained by the impacts of restrictions on fishing effort (Kemp et al. 2020). Importantly, the ongoing effects of Brexit on trade have the potential to impact local seafood availability and the profitability of UK fishing and aquaculture industries.

### Global trade and consumption habits

The UK’s increased reliance on imports is representative of many developed nations around the world (Ye and Gutierrez 2017). Globally, fisheries landings by developed countries have declined, whilst landings by developing countries have continued to rise (Ye and Gutierrez 2017). Furthermore, global aquaculture production has risen dramatically since the 1990s, currently surpassing wild fish capture by more than 18 million tonnes, and over 95% is produced in developing countries (Tacon 2020). In the EU, domestic landings of many demersal fish species have declined since the late 1980s due to stock collapses and restricted distant-water fisheries (Mulazzani and Malorgio 2014). The EU and US have taken significant steps to reduce fishing in local waters to allow stock recovery ﻿(Helvey et al. 2017; Marchal et al. 2016). However, while doing so, they have also significantly increased imports to satisfy consumer demand. As observed in the UK, EU consumer preferences have not adapted to locally available species, with hake and cod being imported in large quantities to compensate for declines in domestic landings (Mulazzani and Malorgio 2014). Similarly, the US imports 90% of its seafood, placing increased environmental pressures on exporting countries (Helvey et al. 2017). Collectively, this has resulted in sharp rises in imports from developing countries to developed countries, with the trade deficit for developed countries doubling since the late 1980s (Ye and Gutierrez 2017).

Despite their reliance on imports to meet current seafood demand, many developed countries are encouraging higher levels of seafood consumption due to the health benefits of eating seafood such as high nutrient and omega-3 fatty acid content (Thurstan and Roberts 2014). This has important health implications in a malnourished world, as many people in the main exporting countries rely on seafood protein to survive (Thurstan and Roberts 2014). It is therefore important to consider ways of reducing the widening gap between seafood supply and consumer demand as we explore below.

### Future directions—reducing the gap between supply and demand

#### Consuming local species

While high fishing pressure can result in stock collapse (Worm et al. 2009), promoting the consumption of underused, locally-caught species through improved marketing and product development could reduce some of the negative impacts of global trade (Robinson et al. 2022; Zhou et al. 2015). In the UK, strong consumer preference for low-landed seafood such as cod, haddock, and tuna is a major driver for the high levels of imports observed today. Increased consumption of species such as mackerel and herring would greatly reduce the gap between supply and demand, however previous attempts to change consumer preferences (e.g. Sainsbury’s “Switch the Fish” and Channel 4’s “Fish Fight” campaign) have had limited success, underscoring the need for further research in this area (Jennings et al. 2016; Urquhart and Acott 2013). Future efforts to promote locally landed species should take advantage of the growing interest in UK food security following Brexit, and more broadly in the importance of sustainable seafood (d’Angelo et al. 2020; Zander and Feucht 2018).

#### Increasing the sustainability of aquaculture production

As wild capture fisheries become increasingly unable to meet seafood requirements due to global population growth, aquaculture production is projected to become the main option to meet consumer demand (Froehlich et al. 2021). In the UK, salmon aquaculture has risen dramatically but farming of other seafood species with high consumer demand is low (Fig. 5). Whilst there have been efforts to promote aquaculture production of other seafood species in the UK, such as cod, haddock, and tilapia, high running costs and other factors have prevented most species from being farmed in large quantities domestically (The Fish Site 2012; Towers 2012; Treasurer 2008). For example, as the cost of rearing cod in aquaculture facilities is higher than catching or importing them from capture fisheries, farmed cod cannot outcompete wild cod on the market (Carrell 2008; Nardi et al. 2021). Globally, aquaculture-produced seafood is exported at a lower rate than wild seafood (Belton et al. 2018), showing potential benefits for reducing carbon emissions (Parker et al. 2018). However, these benefits need to be weighed up against potential environmental costs. Aquaculture is often linked to large-scale habitat loss and degradation through direct alteration of the physical environment (Hamilton et al. 2013; Polidoro et al. 2010) and indirectly through release of excessive nutrients and organic material (Loya et al. 2004; Waycott et al. 2009). The impacts of aquaculture on coral reef, seagrass and mangrove habitats can also affect wild fisheries given their importance as nursery grounds (Clavelle et al. 2019). Finally, the use of wild fish and terrestrial plants in fish feed can place increased pressure on pelagic fisheries (Tacon & Metian 2008) and land-based systems (Naylor et al. 2021), respectively. However, there have been recent promising trends, with the use of wild fish in fish feed decreasing through time (Naylor et al. 2021) and increasing investment into more sustainable feed sources such as microalgae, bacteria, and insects (Cottrell et al. 2020). Aquaculture practices are also being improved, with growing interest in integrated multi-trophic systems and combining aquaculture with wind farms to reduce spatial impacts (Clavelle et al. 2019). Governments should consider the importance of improving aquaculture sustainability and efficiency, and to encourage the consumption of responsibly farmed products.

#### Consuming byproducts & alternative sources of micronutrients

Fish processing accounts for more than 50% loss of landed finfish weight in the UK (Thurstan and Roberts 2014). While only a hypothetical scenario, if it were possible to consume the entirety of gutted fish, UK domestic fish production would meet current seafood demand (Fig. 2a). Fish byproducts such as heads, frames, and livers can be used for direct human consumption but are often discarded or used for fish meal and oil production (Olsen et al. 2014). The meat from byproducts is a good source of protein, minerals, and vitamins, and some countries consume large amounts (Stevens et al. 2018). For example, Norway and Iceland dry and export cod heads in large quantities to Africa, whilst roes and livers are often used for domestic consumption (Olsen et al. 2014).

Another way to reduce pressure on fish stocks whilst meeting health objectives is to encourage the consumption of alternative sources of vitamins, minerals, protein, and omega-3 fatty acids such as algae, flaxseeds, and walnuts (Alcorta et al. 2021; Lenihan-Geels and Bishop 2016; Santos et al. 2020). Given the environmental implications associated with increasing seafood trade and/or production to meet government guidelines, it would be prudent to explore whether the recommendations need to be updated and diversified (Lofstedt et al. 2021; Thurstan and Roberts 2014) and to consider strategies to encourage the consumption of byproducts, to increase food security and reduce pressure on marine resources.

### Study limitations

Unlike other studies that used seafood purchases or surveys to determine consumption (Jennings et al. 2016; Maguire and Monsivais 2015), we estimated consumption indirectly (assuming all UK imports and processed landings and aquaculture that were not exported were consumed domestically). We also converted landed and aquaculture weights to processed weights using static conversion factors, which could result in some inconsistencies in resulting consumption estimates. As we excluded fish meals and oils from the import and export data, all of the traded fish in this study were likely consumed, but some landings may have been used for aquaculture and agriculture feed rather than directly consumed (Tacon and Metian 2008). However, this is likely to be negligible, as most of the fish meal and oil used in the UK is imported (Green 2012). Additionally, the majority of fish meal and oil produced by UK production come from fish trimmings, which were accounted for by calculating processed weight (Green 2012).

A further consideration is that reimported and re-exported fish were not accounted for in this study, so imports and exports may not be entirely independent (Jennings et al. 2016). For example, the data suggests that the UK exported more than 100% of the cod landed in the early 2000s, which is likely due to reimports and re-exports (Fig. 5a). Efforts were made to estimate re-imports and re-exports, but these data are not readily available.

## Conclusion

Over the last 50 years, a combination of major policy changes and strong consumer preferences have resulted in widening gaps between UK seafood production and local consumption, specifically in terms of total weight, species composition and net income. Today, most of the UK’s domestic production is exported and the majority of seafood consumed by the UK public is imported, which may not be environmentally or economically sustainable. In a period of increasing food prices and growing political and environmental instability (Hasegawa et al. 2021; Wheeler and Von Braun 2013), this reliance on imports has serious health, economic and food security implications. This is particularly relevant to the UK’s current situation, as Brexit and recent carbon emission targets will have a large impact on international trade (Stewart 2022; UK Government 2021). However, it is important to consider these impacts on a global level. To avoid overexploiting local fish stocks, many developed nations are meeting consumer demand through imports from developing countries, resulting in environmental and food security ‘cascades’. Clearly, the issues surrounding the international food trade are multifaceted, but it is critical for developed countries to consider environmental, social, economic and health impacts, both at home and overseas.

Collaboration between interdisciplinary researchers, stakeholders and policymakers is essential to ensure that seafood production is sustainable and can meet the requirements of a growing human population. Designing innovative and scientifically informed strategies to increase aquaculture production while reducing environmental impacts should be prioritised. Governments should also consider new approaches to encourage consumers to eat more locally caught and underused species, and promoting seafood byproducts and non-seafood alternatives to increase food production and efficiency. In the face of growing climate and political instability, governments should consider the health, environmental, and economic impacts of increased seafood trade, at both a local and global level.

## Data Availability

The datasets generated and used for the UK seafood production and trade analyses and to produce Figs. 1–6 are available on GitHub at https://github.com/lukeojharrison/UKSeafoodProductionConsumerDemandPaper (Harrison et al. 2023).

## Supplementary ﻿Information

Below is the link to the electronic supplementary material. Supplementary file1 (DOCX 21 KB)
