# Supplementary material for: Widening mismatch between UK seafood production and consumer demand: a 120-year perspective
Source: Rev Fish Biol Fish. 2023 Jun 2:1–22. Online ahead of print. doi: 10.1007/s11160-023-09776-5 (PMC10234684; doi:10.1007/s11160-023-09776-5)
Supplement: Supplementary file 1 — Supplementary file1 (DOCX 21 KB) [file 11160_2023_9776_MOESM1_ESM.docx]

**Table S1.** Data sources for UK fish landings, imports, exports, aquaculture, and population.

| **Data** | **Country** | **Years** | **Source** | **URL** | **Access date** |
| --- | --- | --- | --- | --- | --- |
| Domestic landings, imports, exports, and landings abroad (from 1988) (UK Sea Fisheries Statistics) | UK | 2012-2020 | Marine Management Organisation (MMO) | <https://www.gov.uk/government/collections/uk-sea-fisheries-annual-statistics> | 05/10/2021 |
|  |  | 2009-2011 | Marine Management Organisation (MMO) | [https://webarchive.nationalarchives.gov.uk/20140508034354/http://www.marinemanagement.org.uk/fisheries/statistics/annual_archive.htm](https://webarchive.nationalarchives.gov.uk/20140508034354/http:/www.marinemanagement.org.uk/fisheries/statistics/annual_archive.htm) | 29/07/2021 |
|  |  | 1999-2008 | Department for Environment, Food and Rural Affairs (DEFRA) |  |  |
|  |  | 1965-1998 | Ministry of Agriculture, Fisheries and Food (MAFF) |  |  |
| Domestic landings, imports, and exports (UK Sea Fisheries Statistics) | Great Britain landings, UK trade | 1955-1964 | Ministry of Agriculture, Fisheries and Food (MAFF) | [https://webarchive.nationalarchives.gov.uk/20140508034354/http://www.marinemanagement.org.uk/fisheries/statistics/annual_archive.htm](https://webarchive.nationalarchives.gov.uk/20140508034354/http:/www.marinemanagement.org.uk/fisheries/statistics/annual_archive.htm) | 29/07/2021 |
|  |  | 1922-1954 | Ministry of Agriculture and Fisheries (MAF) |  |  |
| Domestic landings, imports, and exports (UK Sea Fisheries Statistics) | UK & Ireland | 1920-1921 | Ministry of Agriculture and Fisheries (MAF) | [https://webarchive.nationalarchives.gov.uk/20140508034354/http://www.marinemanagement.org.uk/fisheries/statistics/annual_archive.htm](https://webarchive.nationalarchives.gov.uk/20140508034354/http:/www.marinemanagement.org.uk/fisheries/statistics/annual_archive.htm) | 29/07/2021 |
|  |  | 1903–1919 | Board of Agriculture and Fisheries England and Wales |  |  |
|  |  | 1900–1902 | Board of Agriculture and Fisheries United Kingdom and Ireland |  |  |
| Landings in the UK and abroad | UK | 1903-2018 | International Council for the Exploration of the Sea (ICES) | <https://www.ices.dk/data/dataset-collections/Pages/Fish-catch-and-stock-assessment.aspx> | 17/05/2021 |
| UK-based aquaculture production | UK | 1950-2019 | Food and Agriculture Organization (FAO) | <http://www.fao.org/fishery/statistics/global-aquaculture-production/en> | 05/10/2021 |
| UK population | UK (excluding Ireland between 1912-1921) | 1900-2020 | Office of National Statistics (ONS) | <https://www.ons.gov.uk/peoplepopulationandcommunity/populationandmigration/populationestimates/datasets/populationestimatesforukenglandandwalesscotlandandnorthernireland> | 05/10/2021 |
| Ireland population | Ireland | 1912-1921 | Central Statistics Office of Ireland | <https://www.ceicdata.com/en/ireland/population-by-age/estimated-population> | 25/05/2021 |

**Table S2.** Data considerations and manipulations for UK fish landings, imports, exports, aquaculture, and population. All data sources are outlined in Table 1.

| **Data** | **Years** | **Consideration** | **Manipulation (if applicable)** |
| --- | --- | --- | --- |
| Domestic landings, imports, and exports quantity (UK Sea Fisheries Statistics) | 1900-1974 | Fish weight presented in hundredweights (cwt). | Converted to metric tonnes using the standard conversion factor (1 cwt = 0.0508 tonne). |
| Domestic landings quantity (UK Sea Fisheries Statistics) | 1900-1961 | Shellfish landings not available in weight. | ICES landings data was used, which includes domestic landings and landings abroad. Shellfish landings abroad accounted for < 1% of total shellfish landings between 1962-2018 (0.98 % ± SE 0.46 %, n = 57), so their inclusion is unlikely to have a large effect on the main findings. |
| Domestic landings quantity (UK Sea Fisheries Statistics) | 1922-1955 | Landings data only available for Great Britain. | Mean proportion of total landings landed by Northern Ireland between 1930-1955 added to the landings of total fish and individual species (mean = 0.78 % ± SE 0.11 %, n = 26). The proportion was not added to shellfish landings, as ICES landings data was used during this period which already included landings by Northern Ireland. |
| Domestic landings quantity (UK Sea Fisheries Statistics) | 1956-1964 | Mackerel landings data only available for Great Britain. | Mean proportion of total landings landed by Northern Ireland between 1956-1964 added to mackerel landings (mean = 0.93 % ± SE 0.07 %, n = 9). |
| Domestic landings quantity (UK Sea Fisheries Statistics) | 1991-2020 | Reported in Tonnes Live Weight rather than landed weight. | Finfish converted to gutted weight using MMO conversion factors, except herring which are usually landed whole (MMO, 2018). For total finfish, and species without conversion factors, the mean conversion factor for all finfish species except herring was used (mean = 0.89 ± SE 0.01, n = 37). |
| Landings abroad (UK Sea Fisheries Statistics) | 1988-2020 |  |  |
| UK aquaculture production quantity | 1950-2019 |  |  |
| Domestic landings value (UK Sea Fisheries Statistics) | 1922-1955 | Landings value only available for Great Britain. | No action taken. As the mean proportion of total landings landed by Northern Ireland between 1930-1955 was low (mean = 0.78 % ± SE 0.11 %, n = 26), the exclusion of the value of Northern Ireland landings is unlikely to affect the main results. |
| Landings abroad (UK Sea Fisheries Statistics) | 1988-2020 | Landings abroad only available from 1988-2020. | No action taken. |
| Imports quantity (UK Sea Fisheries Statistics) | 1900-1964 | Migratory trout included in salmon imports. | No action taken. |
| Imports quantity (UK Sea Fisheries Statistics) | 1909 | Imports data not available. | Year excluded from analysis. |
| Imports value (UK Sea Fisheries Statistics) | 1900-1950 | Oysters for breeding excluded from total shellfish imports as these are not consumed. | No action taken. |
| Exports quantity (UK Sea Fisheries Statistics) | 1965-1990 | Exports data lacks detail to the species level. | No action taken. |
| Landings in the UK and abroad (ICES catch statistics) | 1922-1930 | Landings by Ireland included despite the partition of Ireland. | No action taken. |
| UK aquaculture production value | 1950-2019 | Reported in USD rather than GBP. | Converted to GBP using annual exchange rates obtained from the Pacific Exchange Rate Service (Antweiler, 2019). |
| UK population | 1900-1953 | Population data by age was not available. | Number of children aged under 15 years old was estimated using the mean proportion between 1953-2019 (20.55 % ± SE 0.29 %, n = 67). |
| UK population | 1912-1921 | Population data for Ireland was not available. | Figures were obtained from the Central Statistics Office of Ireland (CEIC, 2020). |

**References**

Antweiler, W. (2019). *Pacific exchange rate service*. University of British Columbia. Retrieved May 23, 2021, from http://fx.sauder.ubc.ca/etc/USDpages.pdf

CEIC. (2020). *Ireland estimated population*. Computer and Enterprise Investigations Conference Data. Retrieved May 25, 2021, from https://www.ceicdata.com/en/ireland/population-by-age/estimated-population

MMO. (2018). *Conversion factors*. GOV.UK. Retrieved July 29, 2021, from https://www.gov.uk/government/publications/calculate-your-fisheries-catch-limits/conversion-factors
